# Investigating the disordered regions (MoRFs, SLiMs and LCRs) and functions of mimicry proteins/peptides *in silico*

**DOI:** 10.1371/journal.pone.0265657

**Published:** 2022-04-14

**Authors:** Anjali Garg, Govinda Rao Dabburu, Neelja Singhal, Manish Kumar

**Affiliations:** Department of Biophysics, University of Delhi South Campus, New Delhi, India; Russian Academy of Medical Sciences, RUSSIAN FEDERATION

## Abstract

Microbial mimicry of the host proteins/peptides can elicit host auto-reactive T- or B-cells resulting in autoimmune disease(s). Since intrinsically disordered protein regions (IDPRs) are involved in several host cell signaling and PPI networks, molecular mimicry of the IDPRs can help the pathogens in substituting their own proteins in the host cell-signaling and PPI networks and, ultimately hijacking the host cellular machinery. Thus, the present study was conducted to discern the structural disorder and intrinsically disordered protein regions (IDPRs) like, molecular recognition features (MoRFs), short linear motifs (SLiMs), and low complexity regions (LCRs) in the experimentally verified mimicry proteins and peptides (mimitopes) of bacteria, viruses and host. Also, functional characteristics of the mimicry proteins were studied *in silico*. Our results indicated that 78% of the bacterial host mimicry proteins and 45% of the bacterial host mimitopes were moderately/highly disordered while, 73% of the viral host mimicry proteins and 31% of the viral host mimitopes were moderately/highly disordered. Among the pathogens, 27% of the bacterial mimicry proteins and 13% of the bacterial mimitopes were moderately/highly disordered while, 53% of the viral mimicry proteins and 21% of the viral mimitopes were moderately/highly disordered. Though IDPR were frequent in host, bacterial and viral mimicry proteins, only a few mimitopes overlapped with the IDPRs like, MoRFs, SLiMs and LCRs. This suggests that most of the microbes cannot use molecular mimicry to modulate the host PPIs and hijack the host cell machinery. Functional analyses indicated that most of the pathogens exhibited mimicry with the host proteins involved in ion binding and signaling pathways. This is the first report on the disordered regions and functional aspects of experimentally proven host and microbial mimicry proteins.

## Introduction

Microorganisms might exhibit molecular mimicry with the host by displaying similarities either in the sequences/structures of the proteins or, in the short linear motifs of the proteins or, in the structures of the proteins (without sequence homology). The similarities in the microbial peptides and host epitopes might invoke a cross-activation of the host autoreactive T- or B-cells against self-epitopes, resulting in tissue and/or organ destruction and ultimately, autoimmune diseases. The host and the pathogen peptides pairs that show similarity/mimicry with each other are called mimicry peptides or mimitopes.

Several bacteria and viruses like, *Klebsiella pneumoniae*, *Shigella* spp., *Chlamydia trachomatis*, *Helicobacter pylori*, Coxsackievirus, rubella virus etc. have been experimentally implicated in autoimmune diseases like, type I diabetes, multiple sclerosis, autoimmune chronic gastritis, ankylosing spondylitis, reactive arthritis etc [1–5].

Intrinsically disordered proteins (IDPs) and intrinsically disordered protein regions (IDPRs) are those proteins and regions of the proteins respectively, which do not have a well-defined 3D structure. Due to their conformational flexibility, the IDPRs can interact with multiple binding partners, act like hubs in the interaction networks and also perform cellular functions related to signaling and regulation [6–10]. Some conserved functional elements like molecular recognition features (MoRFs) and short linear motifs (SLiMs) have been frequently reported in the IDPRs. MoRFs are amino acid stretches of 10–70 amino acids which are involved in protein-protein interactions (PPIs) and can undergo a disorder-to-order transition upon binding to their partners; this process is termed as coupled folding-binding [11, 12]. IDPs/IDRs can utilize multiple MoRFs simultaneously when interacting with their binding partners [13]. MoRF-containing proteins are present abundantly in the ribosome, nucleus, nucleolus and microtubules and, are involved in translation, protein transport, protein folding, and interactions with DNAs [14]. SLiMs are short stretches of amino acids (3–10 amino acids) which are functionally diverse and mediate signaling interactions [15, 16]. SLiMs regulate the low-affinity interactions and target proteins to a specific subcellular location, recruit enzymes that alter the chemical state of the motif by post-translational modifications, control the stability of a protein, and promote recruitment of binding factors to facilitate complex formation [15, 17]. The short length characteristics of motifs suggest that they might have a high propensity to evolve convergently and emerge in unrelated proteins. Consequently, most of the pathogenic viruses and bacteria evolved to mimic these short linear motifs of the host, allowing them to manipulate cellular processes [18, 19].

Several studies have indicated that viruses manipulate the host cellular machinery by mimicking MoRFs and SLiMs of the host proteins [20, 21]. Similarly, several pathogenic bacteria also mimic the host SLiMs for propagation and sustenance inside the host [22]. Besides MoRFs and SLiMs, disordered regions are also characterized by the presence of low complexity regions (LCRs) which are homo-polymeric repeats of a single amino acid or hetero-polymeric short repeats of a few amino acids residues [23]. Initially, LCRs were considered to be primarily disordered but a few recent studies suggest that LCRs can have a regular secondary structure, also [24]. The LCRs are also associated with important functions like modulation of protein–protein interactions [25], protein–nucleic acid interactions [26], protein subcellular localization [27], antigen processing and diversification [28]. Collectively MoRFs, SLiMs and LCRs facilitate the proteins in adopting several dynamic functional structures, which enables their interaction with multiple binding partners [29]. A few researchers have reported the physico-chemical and structural characteristics of some viral mimicry proteins [20, 21]. However, the presence of disordered regions like MoRFs, SLiMs and LCRs has not been studied in experimentally verified mimicry proteins and mimitopes associated with autoimmune diseases. Since IDPRs are involved in a variety of cell signaling and PPI networks, mimicry of these regions enables the pathogen in substituting its’ own proteins in the host PPI networks and eventually hijack the host cellular machinery. Thus, in the present study we have discerned the presence of order/disorderliness, MoRFs, SLiMs and LCRs in mimicry proteins and mimitopes of bacteria, viruses and host using an *in silico* approach. Additionally, the functional annotation of the mimicry proteins was performed using the Gene Ontology (GO) annotations retrieved from the Gene Ontology Consortium [30].

## Material and methods

### Retrieval of experimentally validated mimicry proteins from miPepBase

The information about bacterial and viral mimicry proteins along with the host mimicry proteins was retrieved from a database of experimentally verified mimicry proteins, miPepBase [31]. The mimicry proteins of the host and pathogen were named as host-protein and pathogen-protein, respectively while, the mimicry peptides (mimitopes) of the host and pathogen were named as host-mimitope and pathogen-mimitope, respectively.

### Order/disorder propensity of amino acids in the host and pathogen mimicry proteins

The order/disorder predisposition of amino acids in the host- and pathogen-proteins was predicted using consensus of three different disorder predictors namely, DISOPRED (version 3.16), IUPred (version 1.0) and PONDER VSL2. DISOPRED is a hybrid predictor based on SVM, neural network and nearest neighbor classifiers [32]. For each amino acid, DISOPRED gives a score between 0–1, with 0.50 as the threshold boundary. Amino acids with a DISOPRED score of ≤0.50 were considered as ordered while with a score >0.50 as disordered. IUPred predicts the disordered/unstructured regions in a protein sequence based on total pairwise inter-residue interaction energy and on the assumption that intrinsically unstructured protein sequences do not fold due to their inability to form a sufficient number of stabilizing inter-residue interactions [33]. Similar to DISOPRED, the score of IUPred prediction also ranges from 0 (complete order) to 1 (complete disorder) with a score above 0.5 indicating disorder. PONDR® FIT (Predictor of Natural Disordered Regions) includes six different types of predictors. We used PONDR® VSL2 since it is considered as the most accurate form of PONDR [34]. In PONDR, an amino acid with a score ≥ 0.5 is considered as disordered. In the present study, all three IDPR predictors were used at default parameters. To address the variability in the predictions of the three predictors, the final disorder predisposition of each amino acid was calculated based on the consensus in all the three predictors. An amino acid was annotated ordered/disordered based on consensus in predictions of at least two of the three predictors. On the basis of consensus prediction, we calculated the percentage of disordered residues (PDR) in a protein by dividing the number of residues in a protein that were predicted as disordered by the total number of residues in that protein. On the basis of PDR, all pathogen- and host-proteins were divided into three categories: highly ordered (PDR < 10%), moderately disordered (10% ≤ PDR < 30%), and highly disordered (PDR ≥ 30%). Previously, several researchers have also used PDR to classify proteins as ordered/disordered [26–29].

### MoRFs, SLiMs and LCRs in the mimicry proteins and mimitopes

The presence of MoRFs in the mimicry proteins and mimitopes was investigated using the MoRFchibi SYSTEM which contains three different modes of MoRF predictions, MoRFCHiBi, MoRFCHiBi_Light, and MoRFCHiBi_Web [35]. In the present study, MoRFCHiBi_Web mode was used, which though slower than the other two modes, gives highly accurate predictions [35].

To predict SLiMs in the mimicry proteins and mimitopes ANCHOR [36] was used and SEG was used to find LCRs [23]. ANCHOR is one very popular tool to predict the protein binding regions in disordered regions of proteins. It has been used in a large number of work to predict the protein-protein binding motif/SLiM regions in a protein [37–45]. Hence in this work we have used ANCHOR to identify binding motifs present in IDP sequences i.e. SLiM. For annotating LCRs in a protein sequence, SEG uses three numeric parameters *viz*. window length (L), trigger complexity (K1), and extension complexity (K2). The whole process of LCR identification by SEG undergoes in two-steps. First, it identifies a low complexity segment using a sliding window of length L amino acids with a local sequence complexity K1. Then all overlapping subsequences with sequence complexity K1 are merged in both directions till the complexity of a contig built by overlapping subsequences does not exceed K2. In the present work, MoRFCHiBi_Web, ANCHOR and SEG were used at default parameters. MoRFs/SLiMs/LCRs were considered to be present within the host or pathogen mimitopes if at least half of the amino acids of the mimitopes overlapped with these regions.

### Functional enrichment analysis

We performed the GO term based functional enrichment analysis of the host and pathogen mimicry proteins using GO mapping tools, OWLTools’ Map2Slim (M2S) (https://github.com/owlcollab/owltools). The functional enrichment analysis was performed at all three levels of the GO annotations namely, Molecular Function, Cellular Component and Biological Process.

## Results

### Benchmarking dataset

A total of 147 bacterial mimicry proteins with corresponding 27 host mimicry proteins (named as Bacterial-set) and, 34 viral mimicry proteins with corresponding 22 host mimicry proteins (named as Viral-set) were retrieved from the miPepBase. The bacterial mimicry proteins were involved in 16 while, viral mimicry proteins were involved in 12 different types of autoimmune diseases. The detailed information about the host and the pathogen, UniProtKB ID and name of the mimicry-protein(s), mimitope sequences, associated autoimmune diseases and the scientific literature (PubMed ID) is summarized in S1 Table.

### Structural order/disorderliness in the mimicry proteins

To assess the extent of structural disorderliness, the mimicry proteins were divided in three different categories based on the PDR, as was also done in earlier studies [26, 27, 41]. Our results revealed that 107 of the 147 bacterial (73%) and 16 of the 34 (47%) viral mimicry proteins were ordered (PDR <10%). This implies that 40 of the 147 (27%) of the bacterial and 18 of the 34 (53%) of the viral mimicry proteins were disordered.

In the Bacterial-set, 21 of the 27 (78%) of the host mimicry proteins and 18 of 40 (45%) of the host mimitopes and, 40 of the 147 (27%) of the bacterial mimicry proteins and 20 of 152 (14%) of the bacterial mimitopes exhibited moderate to high disorderliness.

In the Viral-set 16 of the 22 (73%) of host mimicry proteins and 11 of 35 (31%) of the host mimitopes and, 18 of the 34 (53%) of viral mimicry proteins and 9 of 43 (21%) of the viral mimitopes were moderately/highly disordered (Table 1). The list of proteins that belonged to each category is shown in S2 Table.

**Table 1:** Categorization of mimicry proteins of pathogens and hosts on the basis of PDR (percentage of disordered residues in protein).

Mimicry proteins		Categorization of mimicry proteins on the basis of PDR			No. of mimitopes mapped
		No. of ordered mimicry proteins (PDR <10%)	Number of moderately disordered mimicry proteins (10% ≤ PDR < 30%)	No. of disordered mimicry proteins (PDR ≥ 30%)	
Bacterial-set	Bacterial proteins	107 (73%)	29 (20%)	11 (7%)	20 (14%)
	Host proteins	6 (22%)	8 (30%)	13 (48%)	18 (45%)
Viral-set	Viral proteins	16 (47%)	8 (24%)	10 (29%)	9 (21%)
	Host proteins	6 (27%)	8 (36%)	8 (36%)	11 (31%)

* percent in parentheses depicts the ratio of the number of proteins in each category to the total number of proteins in each dataset.

### MoRFs, SLiMs and LCRs in mimicry proteins/peptides

In the Bacterial-set, 20 of the 27 (74%) host mimicry proteins and 106 of the 147 (72%) bacterial proteins contain MoRFs. In the Viral-set, 13 of the 22 (59%) host mimicry proteins and 32 of the 34 (94%) viral mimicry proteins had MoRFs. With regards to the mimitopes, 7 of 152 bacterial (5%) and 5 of 40 host (13%) mimitopes of the Bacterial-set and, 3 of 43 viral (7%) and 6 of 35 host (17%) mimitopes of the Viral-set overlapped with the MoRFs (Table 2).

**Table 2:** Distribution of MoRFs, SLiMs and LCRs in the mimicry proteins and mimitopes of bacteria, viruses and hosts.

Structural feature	Categorization of mimicry proteins		Number of mimicry proteins in which MoRFs, SLiMs and LCRs were present	Number of proteins in which MoRFs, SLiMs and LCRs were present in mimitope region
Molecular recognition features (MoRFs)	Bacterial-set proteins	Bacterial proteins	106	7
		Host proteins	20	5
	Viral-set proteins	Viral proteins	32	3
		Host proteins	13	6
Short linear motifs (SLiMs)	Bacterial-set proteins	Bacterial proteins	41	13
		Host proteins	16	12
	Viral-set proteins	Viral proteins	20	4
		Host proteins	11	8
Low complexity regions (LCRs)	Bacterial-set proteins	Bacterial proteins	87	8
		Host proteins	19	2
	Viral-set proteins	Viral proteins	30	6
		Host proteins	16	3

Analysis of the SLiM regions in the Bacterial-set revealed that 16 of the 27 (59%) host and 41 of the 147 (28%) of the bacterial mimicry proteins had SLiMs. In the Viral-set, 11 of the 22 (50%) of the host and 20 of the 34 (59%) of the viral mimicry proteins had SLiMs. With regards to the mimitopes, 13 of 152 bacterial (9%) and 12 of 40 host (30%) mimitopes of the Bacterial-set and, 4 of 43 (9%) viral and 8 of 35 (23%) host mimitopes of the Viral-set overlapped with the SLiMs.

In the Bacterial-set, 19 of the 27 host (70%) and 87 of the 147 bacteria (59%) mimicry proteins harbored LCRs. In the Viral-set, 16 of the 22 (73%) host and 30 of the 34 viral (88%) mimicry proteins harbored LCRs. With regards to the mimitopes, 8 of the 152 (5%) bacterial and 2 of 40 (5%) host mimitopes of the Bacterial-set while, 6 of the 43 (14%) viral and 3 of 35 (9%) host mimitopes of the viral-set overlapped with the LCRs (Table 2). The list of proteins that contain MoRF, SLiMs, and LCRs is shown in S2 Table and their number with position in each mimicry-protein is shown in S3 Table.

### Functional characterization of the host and pathogen mimicry proteins

The GO annotations revealed that the host mimicry proteins of the Bacterial-set were involved in biological processes like, anatomical structure development (10 proteins), ion binding (10 proteins), response to stress (7 proteins), immune system process (6 proteins), signal transduction (6 proteins), cell differentiation (5 proteins), cellular protein modification (5 proteins) and protein transport (5 proteins). The host proteins of the Viral-set were involved in like ion binding (9 proteins), anatomical structure development (8 proteins), biosynthetic process (8 proteins), immune system process (7 proteins), signal transduction (7 proteins), cell-cell signaling (7 proteins) and catabolic process (7 proteins) (S4 Table). The bacterial proteins were involved in ion binding (45 proteins), biosynthetic process (31 proteins), cellular nitrogen compound metabolic process (22 proteins), carbohydrate metabolism (16 proteins), transmembrane transport (13 proteins), DNA binding (11 proteins) and DNA metabolic process (10 proteins). Functional enrichment of the viral proteins suggested that most of the viral proteins were a part of symbiont process (28 proteins), cellular nitrogen compound metabolic process (11 proteins), biosynthetic process (10 proteins), DNA binding (10 proteins), ion binding (8 proteins), immune system process (7 proteins) and membrane organization (7 proteins) (S4 Table).

In the molecular function category, the functions of the bacterial and viral host proteins were similar, except that the host proteins of the Viral-set did not perform a few functions like transmembrane transporter activity (S4 Table). The bacterial proteins were involved in molecular functions like ion binding (47 proteins), DNA binding (11 proteins), transmembrane transporter activity (10 proteins). The viral proteins were most prominently involved in symbiont process (28 proteins) followed by DNA binding and structural molecule activity (10 proteins in each function).

**Fig 1:**
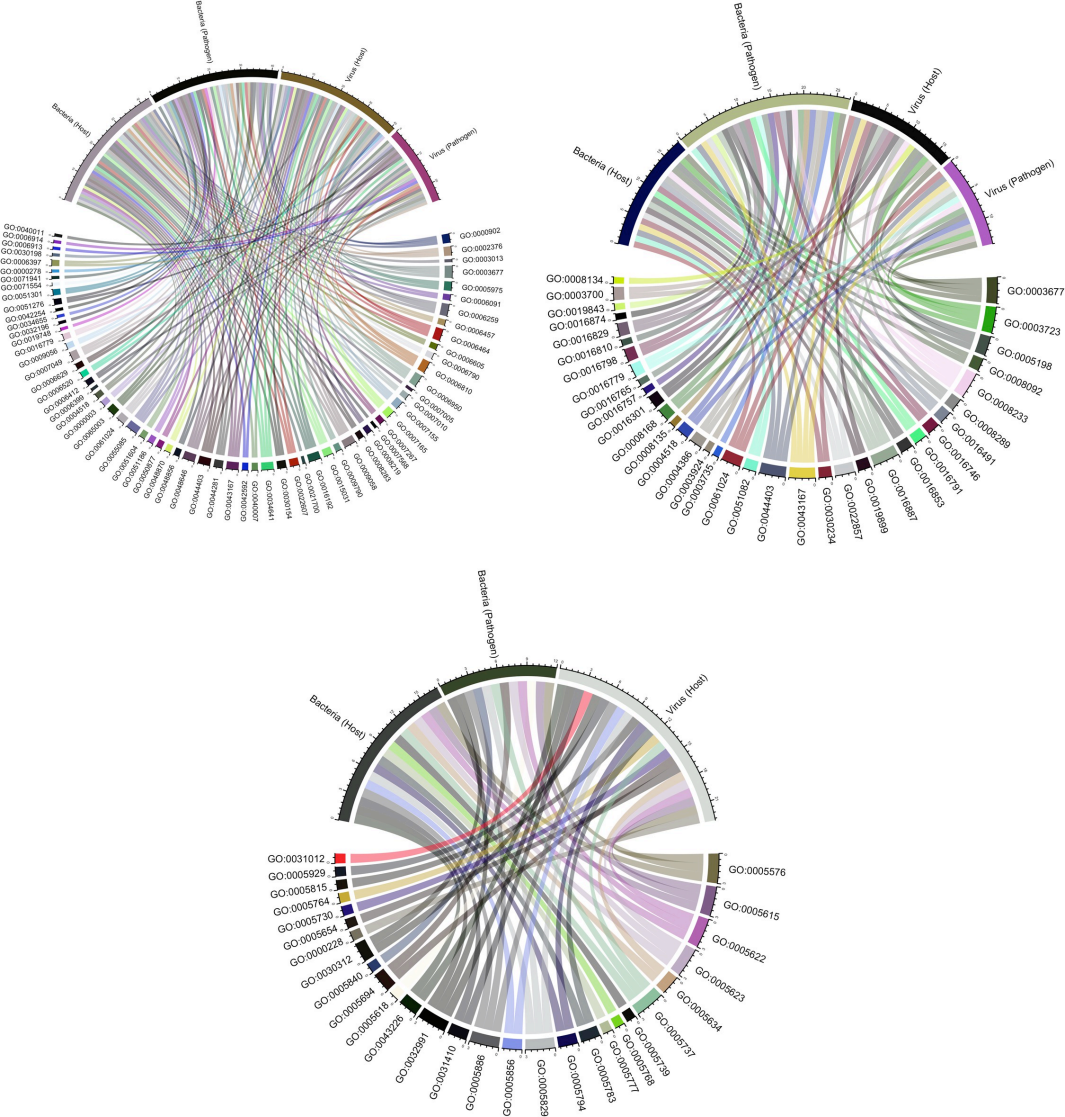
Chord graph representation of gene ontology based functional annotation of bacterial, viral and host mimicry proteins. The GO-term included for: (a) Biological Process, (b) Molecular Function and (c) Cellular Component. Each set is represented by a specific color.

In the category cellular components, the host mimicry proteins of the Bacterial-set were present in protein-containing complex (10 proteins), plasma membrane (9 proteins), and cell (9 proteins), while the bacterial mimicry-proteins showed a significant presence in cytoplasm (28 proteins) followed by plasma membrane (15 proteins) and protein-containing complex (9 proteins). The functional analysis of Viral-set proteins revealed that host mimicry-proteins were localized at the plasma membrane (13 proteins), cell (10 proteins), and cytoplasm (7 proteins) while no specific subcellular enrichment was observed for viral mimicry-proteins (S4 Table). The biological processes, molecular functions and cellular components enriched in each dataset are shown in Fig 1A–1C.

## Discussion

Autoimmune diseases can develop by several mechanisms like, non-specific bystander activation, persistent antigenic stimuli, breach in the central tolerance, host genetics etc [42]. However, microbial molecular mimicry has been recognized as one of the primary mechanisms of pathogen induced autoimmunity. Several studies have indicated that many bacteria and viruses exhibit molecular mimicry with the host proteins and, hijack the host cellular machinery by substituting their own proteins in the host cell signaling and PPI networks [22, 46–49]. Though, the overall fraction of the disordered residues varies in both bacterial and viral proteomes, frequently at least one of the two interacting proteins in viral and host PPIs is structurally disordered [50]. Since, IDPRs are involved in a variety of cell signaling and PPI networks the present study was conducted to discern if the experimentally verified microbial mimicry proteins/mimitopes harbor IDPRs and can thus potentially modulate the host PPIs.

Our results revealed that 78% of the host mimicry proteins and 45% of the host mimitopes involved in bacterial mimicry exhibited moderate to high disorder. Also, 73% of the host proteins and 31% of the host mimitopes involved in viral mimicry exhibited moderate to high disorder. Since disordered regions of the proteins are highly flexible, their presence in the host mimicry proteins might confer a selective advantage to the host in combating the pathogens [51]. With regards to the pathogen, 27% of the bacterial mimicry proteins and 13% of the bacterial mimitopes exhibited moderate to high disorder. In viruses, 53% of the viral mimicry proteins and 21% of the viral mimitopes exhibited moderate to high disorder. Reportedly, the structural disorderliness in viral proteomes might range from as low as 7% in the human coronavirus NL63 to as high as 77% in the avian carcinoma virus, while in bacterial proteomes it usually varies in a small range of 18–35% [51]. As reported in earlier studies, a similar pattern of structural disorderliness was observed in our study, too. The number of viral mimicry proteins and mimitopes which exhibited disorderliness was much greater than bacterial mimicry proteins and mimitopes.

Analysis of the MoRFs, SLiMs and LCRs in the host and microbial mimicry proteins revealed that these regions were present in both the host and microbial mimicry proteins. However, only a few microbial mimitopes overlapped with these regions. The IDPRs like MoRFs, SLiMs and LCRs are frequently involved in various host processes like, cell signaling and PPI interactions [29]. Substitution of the microbial proteins in the host PPI networks by mimicking these regions can greatly facilitate microbial survival and modulation of the host defense mechanisms. On the contrary, our results indicated that only a few microbial mimitopes overlapped with the IDPRs. This suggests that most of the microbial mimitopes experimentally implicated in autoimmune diseases could not potentially modulate functions of the host proteins.

GO-based functional annotation of the host mimicry proteins of the Bacterial- and Viral-set revealed that most of these proteins were multifunctional. For instance, molecular mimicry between the human Zinc transporter 8 protein (Uniprot Id: Q8IWU4) and MAP_3865c protein of *Mycobacterium avium* subsp. *paratuberculosis* results in Type 1 diabetes mellitus. GO-term analysis of the host mimicry-protein Q8IWU4 revealed its involvement in multiple biological processes like ion binding, stress response, immune system process, protein transport, homeostatic process, transmembrane transport, vesicle-mediated transport and cell-cell signaling. This might also be a probable reason underlying its localization at different organelles like, plasma membrane, golgi apparatus and cytoplasmic vesicles (S4 Table). Additionally, many mimicry proteins of the host were involved in ion-binding, DNA/RNA-binding and signaling pathways. It is well-known that metal-sequestering host-defense proteins and microbial metal acquisition machinery are important players in bacterial pathology and disease outcomes [49]. The host immune system counteracts the bacterial infections by reducing metal availability, but the pathogen outmaneuvers this by hijacking the host metalloproteins. In the present study too, we observed that the bacteria and viruses exhibited molecular mimicry with those host proteins that were mainly involved in ion-binding, DNA/RNA-binding and signaling pathways.

## Conclusion

Our results indicated that 78% of the host proteins and 45% of the host mimitopes mimicked by the bacteria were disordered. Also, 73% of the host proteins and 31% of the host mimitopes mimicked by viruses were disordered. In viruses, 53% of the mimicry proteins and 21% of the mimitopes were disordered and in bacteria, only 27% of the mimicry proteins and 13% of the mimitopes were disordered. Moreover, only a few microbial mimitopes overlapped with the IDPRs like MoRFs, SLiMs and LCRs. This suggests that most of the microbial mimitopes experimentally implicated in autoimmune diseases might not potentially modulate the functions of the host proteins. Functional analyses indicated that the host mimicry proteins were mostly involved in ion binding, DNA/RNA-binding and signaling pathways, reflecting that pathogens might preferentially mimic the host proteins which are multi-functional. This is the first *in silico* study investigating the disordered regions and functional aspects of bacterial, viral and host mimicry proteins. We hope our study might serve as a useful platform for further studies on pathogen induced molecular mimicry.

## Supporting information

S1 Table Detailed information about mimicry proteins, peptides and the associated autoimmune diseases of bacteria, viruses and hosts. The data was obtained from miPepBase database (Garg et al. (2016), Frontiers in Microbiology).
(XLSX)

S2 Table Bacterial and Viral-set host and pathogen mimicry proteins containing IDPRs, SLiMs, MoRFs, and LCR. Proteins in which mimicry epitope overlapped with the IDPRs, SLiMs, MoRFs, and LCR are also shown (PDR: Percentage of disordered residues).
(XLSX)

S3 Table The sequence and position (shown in parenthesis) of MoRFs/SLiMs/LCRs into the host and pathogen mimicry proteins. (XLSX)

S4 Table Functional annotation of Bacterial and Viral-set mimicry proteins on the basis of gene ontology (GO) term. The GO-term is mentioned for all three categories namely (i) Biological Process (ii) Molecular Function (iii) Cellular Component.
(XLSX)
